# Occurrence and Characterization of* Salmonella* Isolated from Large-Scale Breeder Farms in Shandong Province, China

**DOI:** 10.1155/2019/8159567

**Published:** 2019-04-16

**Authors:** Jie Yang, Siwei Gao, Yajie Chang, Mingliu Su, Yutong Xie, Shuhong Sun

**Affiliations:** College of Animal Science and Technology, Shandong Agricultural University, Tai'an 271000, China

## Abstract

This study aimed to investigate the prevalence and antimicrobial resistance of* Salmonella* spp. isolated from large-scale breeder farms in Shandong Province, China. A total of 63* Salmonella* isolates (63/409, 15.4%) were identified from 409 samples collected from five large-scale breeder farms in Shandong Province. These* Salmonella* isolates were assayed for serotype, antimicrobial susceptibility, prevalence of class 1 integrons, quinolone resistance genes, and *β*-lactamase genes and subtyped by multilocus sequence typing (MLST). Among these isolates,* S*. Enteritidis (100%) was the predominant serovar, and high antimicrobial resistance rates to nalidixic acid (100.0%), streptomycin (100.0%), ampicillin (98.4%), and erythromycin (93.7%) were observed. All of the isolates carried blaTEM. MLST results showed that only one sequence type (ST11) was identified. Our findings indicated that* Salmonella* was generally prevalent not only on broiler farms but also on breeder farms.

## 1. Introduction

*Salmonella* is the predominant pathogen that causes foodborne illness [1], and it has become one of the most significant zoonoses with many serotypes in recent years. To date, more than 2600 serotypes have been discovered, playing an increasingly important role in disease pathogenesis[2], and the number of people infected with* Salmonella* was higher than that of the summation of several other foodborne diseases in China in 2016 [3].

Currently, there is no effective vaccine for* Salmonella*; however, fractionally feasible measures of* Salmonella* prevention and control that maintain a clean poultry house environment and clean equipment have been adopted, strengthening the management of feeding operations. Antibiotics are the single most effective treatment for* Salmonella*; however, the prevalence of multiple* Salmonella* serovars in the environment has been induced by the extensive use of antibiotics by humans, which creates tremendous potential dangers for the treatment of patients with* Salmonella* infections. The resistance rate of* Salmonella* to Ampicillin (AMP) in the population was 90% in 2018 [4], and resistance is causing a public health threat.

Shandong is one of the highest producers of poultry products. The quantity of poultry heads accounts for 15% of the total number in China, and its products, including eggs and broiler meat, are exported to multiple countries [5]. There are many farms in Shandong, and the scales of farms and types of chickens are different. A lack of published surveys on domestic breeder farms that makes the epidemic situation invisible to the public, the pathogenicity of* Salmonella* in different regions, and the enormous expense and detriment to farms caused by* Salmonella* prompted us to conduct a study of breeder farms in Shandong. In this study, the excrements from 5 large-scale breeder farms were randomly sampled, and genetic species of* Salmonella* were analyzed. Resistance characteristic tests were performed using MLST, and class I integrons were detected.

## 2. Materials and Methods

### 2.1. Sample Collection

From April 2018 to May 2018, a total of 409 samples, including 229 fresh fecal swabs and 180 chicken embryos, were randomly collected from five large-scale breeder farms in Shandong Province (Table 1). Chicken cages were randomly selected for sampling (one sample per cage). All samples were collected from two large-scale breeder farms in Binzhou (n=219), two large-scale breeder farms in Heze (n=120), and a large-scale breeder farm in Liaocheng (n=70). Breeder farms were chosen based on their scale; stock varied from 10,000 to 100,000 chickens. Every sampling site was visited only once. After sampling, samples were immediately transported to our laboratory within 6 h in a box containing ice.

### 2.2. Isolation and Identification of* Salmonella*

The sampling strategy was defined according to the standard method of China GB 4789.4-2010, with some modifications. Briefly, 10.0 mL of buffered peptone water (BPW, Land Bridge Technology, Beijing, China) was added to each sample (1 g) for preenrichment. After incubation at 37°C for 18 h, 1.0 mL preenriched culture was inoculated into 10.0 mL selenite cystine broth (SC, Land Bridge Technology) and incubated at 42°C for 24 h. One loop of each SC broth culture was then streaked onto xylose lysine deoxycholate medium (XLD, Land Bridge Technology) plates and incubated at 37°C for 24 h to 48 h. Next, nonrepetitive suspected* Salmonella* colonies were identified and confirmed by PCR amplification of the invA gene [6].

### 2.3. Salmonella Serotyping

*Salmonella* isolates were serotyped by slide agglutination with O and H antigen-specific sera (Tianrun Bio-Pharmaceutical, Ningbo, China) according to the Kauffmann-White scheme [7].

### 2.4. Antimicrobial Susceptibility Testing

The Kirby-Bauer disk diffusion method, which was learned from the Clinical and Laboratory Standards Institute (CLSI, 2013) [8] (Table 6), was applied to examine the sensitivity of* Salmonella* to 13 commonly used antibiotics, including nalidixic acid (NAL; 30 *μ*g), norfloxacin (NOR;10 *μ*g) erythromycin (EM; 15 *μ*g), streptomycin (STR;10 *μ*g), ampicillin (AMP; 10 *μ*g), tetracycline (TET; 30 *μ*g), ciprofloxacin (CIP; 5 *μ*g), chloramphenicol (CHL; 30 *μ*g), ceftriaxone (CRO; 30 *μ*g), cefotaxime (CTX; 30 *μ*g), gentamicin (GEN; 10 *μ*g), sulfamethoxazole (SXT; 25 *μ*g), and polymyxin B (PB; 300 IU).* Escherichia* coli strains ATCC 25922 and ATCC 35218 were used as the quality control strains.* Salmonella* isolates resistant to more than three classes of antimicrobials were defined as multidrug resistance (MDR) isolates [9, 10]. The AMP belonged to penicillin. The CTX and CRO were from cephalosporin. The GEN and STR were from aminoglycosides. The EM was from macrocyclic lipids. The TET pertained to tetracycline. The PB pertained to polypeptides. The MOR, CIP, and NAL were from quinolone. The SXT pertained to sulfonamides. The CHL was from chloramphenicol.

### 2.5. Detection of Class I Integrons and Resistance Gene

The DNA of positive bacterial strains was extracted using a TIANamp Bacterial DNA Kit (Tiangen, Beijing, China) according to the manufacturer's instructions. The gene cassettes within the variable regions of class I integrons were detected via polymerase chain reaction (PCR) with previously described primers according to previously described procedures [11]. The sequences of primers are shown in Table 2 [12]. The PCR mixture volume was 25 *μ*L. After initial denaturation at 94°C for 10 min, the samples underwent a series of 35 cycles of 1 min denaturation at 94°C, 1 min annealing at 55°C, and 1 min elongation at 72°C. This was followed by a final elongation step at 72°C for 10 min, and samples were stored at 4°C.

*β*-lactamase encoding genes (*bla*_TEM_, *bla*_PSE-1_, *bla*_CMY-2_, *bla*_SHV_, *bla*_OXA_, and *bla*_CTX-M_) and quinolone resistance genes (*qnrA*,* qnrB*,* qnrC*,* qnrD*, and* qnrS*) were detected via PCR using previously described primers and procedures [9, 13, 14]; the sequences of primers are shown in Table 3 [15, 16]. Then, the PCR products were visualized by agarose gel electrophoresis. The PCR mixture volume was 50 *μ*L. After initial denaturation at 94°C for 5 min, the samples underwent a series of 32 cycles of 30 s denaturation at 94°C, 30 s annealing at 55°C, and 45 s elongation at 72°C. This was followed by a final elongation step at 72°C for 7 min, and samples were stored at 4°C.

### 2.6. MLST

According to the description from the University of Warwick (http://mlst.warwick.ac.uk/mlst/), seven housekeeping genes (*aroC, dnaN, hemD, hisD, purE, sucA,* and* thrA*) were used for the molecular typing of* Salmonella* strains. Sequence types (STs) were assigned according to the STs in the* Salmonella* enterica MLST database (http://mlst.warwick.ac.uk/mlst/dbs/Senterica) [17].

## 3. Results

### 3.1. Prevalence and Serotypes of* Salmonella*

A total of 63* Salmonella* strains were isolated from 409 samples obtained from five large-scale breeder farms, for an isolation rate of 15.4%. However, 63 strains were all collected from Binzhou (Table 1). In our study, 63* Salmonella* isolates were divided into one serotype which were* S*. Enteritidis (Table 4).

### 3.2. Antimicrobial Susceptibility Testing

According to the standard of CLSI, all 63* Salmonella* strains were susceptible to NOR, CIP, CHL, GEN, SXT, and PB. All of the isolates were resistant to NAL and STR (Table 7). Most isolates were resistant to AMP (62/63, 98.41%) and EM (59/63, 93.65%). Only one isolate (1/63, 1.59%) was resistant to CRO, and the other isolate (1/63, 1.59%) had tolerance to CTX (Table 5). The most common drug resistance spectrum was AMP-EM-NAL-STR (n=38). In addition, all of the isolates (n=63) exhibited multidrug resistance (MDR). There was resistance to quinolones, tetracyclines, penicillins, cephalosporins, aminoglycosides, and macrolides.

### 3.3. Characteristics of Class I Integrons and Resistance Genes

There were no class I integrons among the 63* Salmonella* strains. Among the 63* Salmonella* strains isolated, blaTEM was the most frequently detected, with a detection rate of 100%. Quinolone resistance genes were not detected.

### 3.4. MLST

Through MLST analysis, only one ST was identified, ST11 (63/63,100%), among the 63 strains. The ST identified in the present study (ST11) correlated with the* Salmonella *serovar* S*. Enteritidis.

## 4. Discussion

Shandong is not only an important producer of domestic poultry products for all parts of country but also one of the most important producing areas for exportation abroad. In Shandong, Liaocheng transported 120 million poultry products to other countries in 2017 [18]. At present, there are few reports on large-scale breeder farms, so it is important to understand the* Salmonella* contamination degree of poultry products in Shandong. According to our study, 63* Salmonella* isolates were identified, with a detection rate of 15.4%, which was lower than the detection rate in Shandong in 2016 (23.4%) [19]. This may be related to the size of the farm, the variety of the chicken, and the environment on the farm. In addition, the detection rate in this study was lower than the detection rate of broiler farms (26.6%) in 2017 [20]. Possibly, the reduction is due to current stricter management of breeder farms. The 63 strains of* Salmonella* were from the same farm. The detection rate of the farm was higher than 23.4% and 26.6%, indicating that there are hidden dangers regarding* Salmonella* contamination. The trends of* Salmonella* prevalence have been increasing in recent years [3], so the farm may also affected by these factors. At the same time, the detection rate of another breeder farm in the same area was 0%, which may be related to the management and sanitation of the breeder farm.

In our research, we found that the dominant serotype was* S*. Enteritidis. However, other relevant research reports showed that* S*. Gallinarum was the dominant serotype in some places [21], and sometimes* S*. Indiana [22] dominated. In different areas, the dominant serotype is different. Although* S*. Typhimurium prevalence is serious [23], we could not examine the serotype as only* S*. Enteritidis responds MLST. This can be relevant to the size and the location of the farm.* S*. Enteritidis abundance was serious on farm 1. Single serotypes may be associated with the kinds of breeder farms.* Salmonella* can be transmitted vertically to infect livestock offspring [24]. In this study, breeders were investigated, and the breeding farm was closed. If parent stock breeders do not transmit* Salmonella*, the probability of infection can be reduced greatly. In contrast, if parent stock is infected, it will give rise to the serious prevalence of* Salmonella*. The dominant serotype was reflected by the parental infection serotype; therefore, the parent stock breeders may have been transmitting* Salmonella* on farm 1. Although the farm was seriously contaminated by* S*. Enteritidis, it was the only serotype present, so infection prevention should be easy, which gives us more motive to take action.

In this study, all* Salmonella* isolates were completely resistant to NAL (100%); in 2017, most were resistant to NAL (99.5%), and resistance was less. AMP resistance increased most significantly compared with the 2017 report. The national AMP resistance (87.8%) rose to 98.4%, indicating that the farm had more applications of AMP, much higher than the Chinese average. In broiler farms in 2017, the antimicrobial resistance to TET and CRO was clearly higher than that of breeder farms [25]. The antimicrobial resistance of NAL and AMP increased slightly, which may be related to time. Antimicrobial resistance has reached high levels in all domestic farms. This may be because the major drugs for the treatment of* Salmonella* are NAL and AMP, and antimicrobial resistance has increased year by year, eventually reaching full drug resistance. The antimicrobial resistance of the breeder farm is relatively lower than that of the broiler farm. This may be because the breeding management of the breeder farm is more stringent, and the medication administration is limited, which reduces the spread of antimicrobial resistance. Moreover, compared with resistance rates in 2011-2014, related research has shown that resistance has increased, showing an increasing trend year to year. For example, STR resistance (100%) was 34.0% in 2011-2014; the increase may be due the excessive use of STR drugs in plants [26]. Compared with NAL (41%) in Sichuan in 2013, present day values are far higher, with resistance increases yearly; however, this may be different from the local epidemic serotype, and the regimen of drugs may be different, resulting in different drug resistance patterns [9].

The result of MLST showed that only one ST, ST11, was found among the isolates from the chicken farms. This is similar to animal-derived* Salmonella* research results from Shandong in 2017 [27]. In addition, ST40 and ST17, which are more common in other research reports, were not detected in this study [28]. This may be because breeder farms may be less contaminated than broiler farms, meat products may be contaminated by* Salmonella*, or* S*. Enteritidis contamination is more serious. If the ST is the same, it means that parent stock transmitted the same* Salmonella* strain. A single ST may be related to the research subject, which consisted of breeding farms in this research. Perhaps the 63* Salmonella* isolates evolved from one* Salmonella* isolate, and the offspring were infected by parent stock; therefore, the possibility of infection from a single* Salmonella* isolate increased greatly. It increases the possibility of the same ST. In this study, ST11 corresponded to* Salmonella* Enteritidis, which is consistent with previous reports [29]. However, some studies have shown that, in America, the major prevalent STs are ST34, ST33, and ST11 [30], which shows that STs may be associated with regions.

Resistance gene detection results only identified one drug resistance gene, blaTEM. There were no blaPSE genes detected. Since the MLST resulted in only one ST, the ST may be from the same generation of* Salmonella*. It is likely that drug resistance genes are also derived from the parents, resulting in the detection of only one resistance gene (100%). In 2014, in chickens with enterointestinal* Salmonella*, the detection rate of the blaTEM resistance gene was greater than 88% [31]. With the widespread use of antibiotics, the frequency of the emergence of resistant genes has also increased. On breeder farms, the transmission of* Salmonella* to offspring resulted in a significant increase in blaTEM gene frequency, which may be responsible for the significantly higher detection rate, up to 100%, than that observed in 2014 as well as that observed in the 2017 Taiwan drug resistance study [32]. The detection rate of other drug resistance genes was low, which may be related to genotype or region. Because of various dominant serotypes and climates in different regions, the results will differ.

What is puzzling about* Salmonella* Gallinarum detected in 2013 is that no gene cassettes were found in the class 1 integrons [33], possibly because all serotypes were ST11.

## 5. Conclusions

Our investigation showed that only one serotype of* Salmonella*,* S*. Enteritidis, was identified in large-scale breeder farms in Shandong. Farms can enhance feeding management and eliminate contaminated parent generation chickens in a timely manner to control* Salmonella* outbreaks. Although different strains of* Salmonella* in the same breeder farm show different drug resistance rates, they all have high resistance to common compounds, and their drug resistance spectrum is also common because of the abuse of antibiotics. The situation is dangerous to public health and safety, and people should consider the responsible use of antibiotics, utilize a combination or rotation of drugs, use less antimicrobial drugs, and be aware of the course of treatment and withdrawal period to ensure our food safety.

## Figures and Tables

**Table 1:** The prevalence of *Salmonella *in chicken farms.

Location	Variety	Sample type	No. of samples	No. of positive samples
Binzhou Farm 1	breeders	chicken embryos	180	63 (35.0%)
Binzhou Farm 2	breeders	fecal swabs	39	0
Heze Farm1	breeders	fecal swabs	60	0
Heze Farm2	breeders	fecal swabs	60	0
Liaocheng Farm1	breeders	fecal swabs	70	0

**Table 2:** Primers of class I.

Primers	Primers' sequences 5'–3'	Product length (bp)
Class1*-*F	TCATGGCTTGTTATGACTGT	856bp
Class1*-*R	GTAGGGCTTATTATGCACGC	

**Table 3:** Primers of resistance genes.

bla_TEM_	F:5′-ATAAAATTCTTGAAGACGAAA-3′	643bp
	R:5′-GACAGTTACCAATGCTTAATC-3′	
bla_SHV_	F:5′-TTATCTCCCTGTTAGCCACC-3′	860bp
	R:5′-GATTTGCTGATTTCGCTCGG-3′	
bla_PSE_	F:5′-TAGGTGTTTCCGTTCTTG-3′	150bp
	R:5′-TCATTTCGCTCTTCCATT-3′	
bla_OXA_	F:5′-TCAACTTTCAAGATCGCA-3′	591bp
	R:5′-GTGTGTTTAGAATGGTGA-3′	
bla_CMY-2_	F:5′-ACGGAACTGATTTCATGATG-3′	714bp
	R:5′-GAAAGGAGGCCCAATATCCT-3′	
bla_CTX-M_	F:5′-CGCTTTGCGATGTGCAG-3′	550bp
	R:5′-ACCGCGATATCGTTGGT-3′	
qnrA	F:5′-ATTTCTCACGCCAGGATTTG-3′	519bp
	R:5′-GATCGGCAAAGGTCAGGTCA-3′	
qnrB	F:5′-GATCGTGAAAGCCAGAAAGG-3′	513bp
	R:5′-ACGATGCCTGGTAGTTGTCC-3′	
qnrC	F:5′-GGTTGTACATTTATTGAATC-3′	666bp
	R:5′-TCCACTTTACGAGGTTCT-3′	
qnrD	F:5′-AGATCAATTTACGGGGAATA-3′	984bp
	R:5′-AACAAGCTGAAGCGCCTG-3′	
qnrS	F:5′-ACGACATTCGTCAACTGCAA-3′	417bp
	R:5′-TAAATTGGCACCCTGTAGGC-3′	

**Table 4:** Resistance phenotype, MLST, and resistance gens in *Salmonella* isolated from breeder farms.

No.	Serovar	MLST	Resistance phenotype	Resistance genes
1	*S*. Enteritidis	ST-11	AMP-EM-NAL-STR	*bla*_TEM_
2	*S*. Enteritidis	ST-11	AMP-EM-NAL-STR-TET	*bla*_TEM_
3	*S*. Enteritidis	ST-11	AMP-NAL-STR	*bla*_TEM_
4	*S*. Enteritidis	ST-11	AMP-NAL-STR-TET	*bla*_TEM_
5	*S*. Enteritidis	ST-11	AMP-EM-NAL-STR	*bla*_TEM_
6	*S*. Enteritidis	ST-11	AMP-EM-NAL-STR-TET	*bla*_TEM_
7	*S*. Enteritidis	ST-11	AMP-EM-NAL-STR	*bla*_TEM_
8	*S*. Enteritidis	ST-11	AMP-EM-NAL-STR	*bla*_TEM_
9	*S*. Enteritidis	ST-11	AMP-EM-NAL-STR	*bla*_TEM_
10	*S*. Enteritidis	ST-11	AMP-EM-NAL-STR	*bla*_TEM_
11	*S*. Enteritidis	ST-11	AMP-EM-NAL-STR-TET	*bla*_TEM_
12	*S*. Enteritidis	ST-11	AMP-EM-NAL-STR	*bla*_TEM_
13	*S*. Enteritidis	ST-11	AMP-EM-NAL-STR	*bla*_TEM_
14	*S*. Enteritidis	ST-11	AMP-EM-NAL-STR-TET	*bla*_TEM_
15	*S*. Enteritidis	ST-11	AMP-EM-NAL-STR	*bla*_TEM_
16	*S*. Enteritidis	ST-11	AMP-EM-NAL-STR-TET	*bla*_TEM_
17	*S*. Enteritidis	ST-11	AMP-CRO-EM-NAL-STR	*bla*_TEM_
18	*S*. Enteritidis	ST-11	AMP-EM-NAL-STR-TET	*bla*_TEM_
19	*S*. Enteritidis	ST-11	AMP-EM-NAL-STR	*bla*_TEM_
20	*S*. Enteritidis	ST-11	AMP-EM-NAL-STR	*bla*_TEM_
21	*S*. Enteritidis	ST-11	AMP-EM-NAL-STR	*bla*_TEM_
22	*S*. Enteritidis	ST-11	AMP-EM-NAL-STR-TET	*bla*_TEM_
23	*S*. Enteritidis	ST-11	AMP-EM-NAL-STR	*bla*_TEM_
24	*S*. Enteritidis	ST-11	AMP-EM-NAL-STR	*bla*_TEM_
25	*S*. Enteritidis	ST-11	AMP-EM-NAL-STR	*bla*_TEM_
26	*S*. Enteritidis	ST-11	AMP-EM-NAL-STR	*bla*_TEM_
27	*S*. Enteritidis	ST-11	AMP-NAL-STR-TET	*bla*_TEM_
28	*S*. Enteritidis	ST-11	AMP-EM-NAL-STR-TET	*bla*_TEM_
29	*S*. Enteritidis	ST-11	AMP-EM-NAL-STR	*bla*_TEM_
30	*S*. Enteritidis	ST-11	AMP-EM-NAL-STR	*bla*_TEM_
31	*S*. Enteritidis	ST-11	AMP-EM-NAL-STR	*bla*_TEM_
32	*S*. Enteritidis	ST-11	AMP-EM-NAL-STR	*bla*_TEM_
33	*S*. Enteritidis	ST-11	AMP-EM-NAL-STR	*bla*_TEM_
34	*S*. Enteritidis	ST-11	AMP-EM-NAL-STR-TET	*bla*_TEM_
35	*S*. Enteritidis	ST-11	AMP-EM-NAL-STR-TET	*bla*_TEM_
36	*S*. Enteritidis	ST-11	AMP-NAL-STR	*bla*_TEM_
37	*S*. Enteritidis	ST-11	AMP-EM-NAL-STR	*bla*_TEM_
38	*S*. Enteritidis	ST-11	AMP-EM-NAL-STR-TET	*bla*_TEM_
39	*S*. Enteritidis	ST-11	AMP-CTX-EM-NAL-STR-TET	*bla*_TEM_
40	*S*. Enteritidis	ST-11	AMP-EM-NAL-STR	*bla*_TEM_
41	*S*. Enteritidis	ST-11	AMP-EM-NAL-STR	*bla*_TEM_
42	*S*. Enteritidis	ST-11	AMP-EM-NAL-STR	*bla*_TEM_
43	*S*. Enteritidis	ST-11	AMP-EM-NAL-STR	*bla*_TEM_
44	*S*. Enteritidis	ST-11	AMP-EM-NAL-STR-TET	*bla*_TEM_
45	*S*. Enteritidis	ST-11	AMP-EM-NAL-STR	*bla*_TEM_
46	*S*. Enteritidis	ST-11	AMP-EM-NAL-STR	*bla*_TEM_
47	*S*. Enteritidis	ST-11	AMP-EM-NAL-STR	*bla*_TEM_
48	*S*. Enteritidis	ST-11	AMP-EM-NAL-STR-TET	*bla*_TEM_
49	*S*. Enteritidis	ST-11	AMP-EM-NAL-STR-TET	*bla*_TEM_
50	*S*. Enteritidis	ST-11	AMP-EM-NAL-STR	*bla*_TEM_
51	*S*. Enteritidis	ST-11	AMP-EM-NAL-STR	*bla*_TEM_
52	*S*. Enteritidis	ST-11	AMP-EM-NAL-STR-TET	*bla*_TEM_
53	*S*. Enteritidis	ST-11	AMP-EM-NAL-STR-TET	*bla*_TEM_
54	*S*. Enteritidis	ST-11	AMP-EM-NAL-STR-TET	*bla*_TEM_
55	*S*. Enteritidis	ST-11	AMP-EM-NAL-STR-TET	*bla*_TEM_
56	*S*. Enteritidis	ST-11	AMP-EM-NAL-STR	*bla*_TEM_
57	*S*. Enteritidis	ST-11	AMP-EM-NAL-STR	*bla*_TEM_
58	*S*. Enteritidis	ST-11	AMP-EM-NAL-STR-TET	*bla*_TEM_
59	*S*. Enteritidis	ST-11	AMP-EM-NAL-STR-TET	*bla*_TEM_
60	*S*. Enteritidis	ST-11	AMP-EM-NAL-STR	*bla*_TEM_
61	*S*. Enteritidis	ST-11	AMP-EM-NAL-STR	*bla*_TEM_
62	*S*. Enteritidis	ST-11	EM-NAL-STR	*bla*_TEM_
63	*S*. Enteritidis	ST-11	AMP-EM-NAL-STR	*bla*_TEM_

(a) ST, sequence type.

(b) Nalidixic acid = NAL; norfloxacin= NOR; erythromycin = EM; streptomycin=STR; ampicillin = AMP; tetracycline = TET; ciprofloxacin= CIP; chloramphenicol= CHL; ceftriaxone = CRO; cefotaxime= CTX; gentamicin = GEN; sulfamethoxazole= SXT; polymyxin =PB.

**Table 5:** standard of CLSI.

	CIP	NAL	EM	STR	MOR	CTX	GEN	AMP	SXT	CRO	PB	TET	CHL
R	≤20	≤13	≤13	≤11	≤12	≤22	≤12	≤13	≤10	≤19	≤8	≤14	≤12
I	20-30	14-18	14-22	12~14	13-16	23-25	13-14	14-16	11~15	20-22	8~11	15-18	13-17
S	≥31	≥19	≥23	≥15	≥17	≥26	≥15	≥17	≥16	≥23	≥12	≥19	≥18

(a) R= resistant; I= intermediate; S= susceptible.

(b) Nalidixic acid = NAL; norfloxacin= NOR; erythromycin = EM; streptomycin=STR; ampicillin = AMP; tetracycline = TET; ciprofloxacin= CIP; chloramphenicol= CHL; ceftriaxone = CRO; cefotaxime= CTX; gentamicin = GEN; sulfamethoxazole= SXT; polymyxin =PB.

**Table 6:** Susceptibility profiles of the in *Salmonella* isolated from breeder farms.

Antibiotic	No. of Salmonella isolates (n* *=* *63)		
	Resistant	Intermediate	Susceptible
Ampicillin (AMP)	62 (98.41%)	0 (0.00%)	1 (1.58%)
Cefotaxime (CTX)	1 (1.59%)	0 (0.00%)	62 (98.41%)
Ceftriaxone (CRO)	1 (1.59%)	1 (1.59%)	61 (96.82%)
Gentamicin (GEN)	0 (0.00%)	0 (0.00%)	63 (100.00%)
Streptomycin (STR)	63 (100.00%)	0 (0.00%)	0 (0.00%)
Erythromycin (EM)	60 (95.24%)	3 (4.76%)	0 (0.00%)
Tetracycline (TET)	23(36.51%)	34 (53.97%)	6 (9.52%)
Polymyxin (PB).	0 (0.00%)	0 (0.00%)	63 (100.00%)
Norfloxacin (NOR)	0 (0.00%)	1 (1.59%)	62 (98.41%)
Ciprofloxacin (CIP)	0 (0.00%)	63 (100.00%)	0 (0.00%)
Nalidixic acid (NAL)	63 (100.00%)	0 (0.00%)	0 (0.00%)
Sulfamethoxazole (SXT)	0 (0.00%)	0 (0.00%)	63 (100.00%)
Chloramphenicol (CHL)	0 (0.00%)	0 (0.00%)	63 (100.00%)

(a) Resistant, the antibiotic may be totally off limits under the circumstances; Intermediate, it implies clinical applicability in body sites where drugs are intermediate categories and also indicates “buffer zone”, which prevent small uncontrolled technical factors from causing major discrepancies, especially for drugs with narrow pharmacotoxicity margins; Susceptible, the infection due to the strain tested may be expected to respond to usual dosage of this antimicrobial agent.

## Data Availability

The data used to support the findings of this study are available from the corresponding author upon request.

## Supplementary Materials

Supplementary Materials Table 7 is the supplementary material, which includes the data of inhibition zone of 63* Salmonella* in drug susceptibility test.
